# Enzyme 15-lipoxygenase 1 promotes hypoxia-inducible factor 1*α* turnover and reduces vascular endothelial growth factor expression: implications for angiogenesis

**DOI:** 10.1002/cam4.227

**Published:** 2014-03-25

**Authors:** Hua Zhong, Ruoxiang Wang, Uddhav Kelavkar, Christopher Y Wang, Jonathan Simons

**Affiliations:** 1 Department of Urology and Winship Cancer Institute, Emory University School of MedicineAtlanta, Georgia, 30322; 2 Rutgers Cancer Institute of New Jersey and Department of Pathology and Laboratory Medicine, Rutgers Robert Wood Johnson Medical SchoolNew Brunswick, New Jersey, 08901; 3 Uro-Oncology Research, Department of Medicine, Cedars-Sinai Medical CenterLos Angeles, California, 90048; 4 Mercer University School of Medicine1550 College Street, Macon, Georgia, 31207; 5 Prostate Cancer Foundation1250 4th Street, Santa Monica, California, 90401

**Keywords:** 15-lipoxygenase 1, HIF-1*α*, hypoxia, ubiquitination, VEGF

## Abstract

Hypoxia-inducible factor 1*α* (HIF-1*α*) is the regulatory subunit of the heterodimeric HIF-1 that plays a critical role in transcriptional regulation of genes in angiogenesis and hypoxic adaptation, while fatty acid metabolism mediated by lipoxygenases has been implicated in a variety of pathogeneses, including cancers. In this study, we report that 15-lipoxygenase 1 (15-LO1), a key member of the lipoxygenase family, promotes HIF-1*α* ubiquitination and degradation. Altering the level of 15-LO1 yields inverse changes in HIF-1*α* and HIF-1 transcriptional activity, under both normoxia and hypoxia, and even in CoCl_2_-treated cells where HIF-1*α* has been artificially elevated. The antagonistic effect of 15-LO1 is mediated by the Pro^564^/hydroxylation/26S proteasome system, while both the enzymatic activity and the intracellular membrane-binding function of 15-LO1 appear to contribute to HIF-1*α* suppression. Our findings provide a novel mechanism for HIF-1*α* regulation, in which oxygen-dependent HIF-1 activity is modulated by an oxygen-insensitive lipid metabolic enzyme.

## Introduction

The role of hypoxia-inducible factor 1*α* (HIF-1*α*) in cellular adaptation to hypoxia has been well established 1. As the regulatory subunit of the heterodimeric HIF-1 transcription factor complex, HIF-1*α* turnover is finely tuned by intracellular and extracellular cues under normoxia to maintain homeostasis. This adaptive mechanism is frequently hijacked for the growth and survival of malignant cells, as evidenced by our previous finding that HIF-1*α* is abnormally activated in most types of cancer 2. With the tropisms of HIF-1*α* on proliferation, migration, and invasion, its activation would be consequential to tumor growth and metastasis. In addition, HIF-1 can activate vascular endothelial growth factor (*VEGF*) gene transcription, which may induce intratumoral angiogenesis, further facilitating tumor growth 1. HIF-1*α* is thus a critical target for the prevention of cancer progression and distant metastasis. Extensive efforts have been devoted to modulate HIF-1*α* so that its oncogenic transcription activity can be targeted for cancer therapy.

Newly synthesized HIF-1*α* is rapidly degraded via the von Hippel-Lindau tumor suppressor (pVHL)-dependent ubiquitin-proteasome pathway 1,3, which is mediated by the hydroxylation of proline residues (Pro402 and Pro564) within the oxygen-dependent degradation (ODD) domain of HIF-1*α* by a group of prolyl hydroxylases (PHDs) 4. Factor-inhibiting HIF-1 (FIH-1) suppresses HIF-1 transactivation through the hydroxylation of an asparaginyl residue on HIF-1*α* 5, thus blocking the association of HIF-1*α* with its coactivator protein p300 6. Under hypoxic conditions, or in cells with pVHL dysfunction, HIF-1*α* escapes PHD-dependent degradation and accumulates intracellularly. Inhibition of PHD by *α*-ketoglutarate (a PHD substrate) antagonist—dimethyloxalyxalyglycine (DMOG), iron chelator or cobalt chloride can interrupt rapid HIF-1*α* degradation in the presence of a normal level of O_2_ 4.

Aside from the key regulators that include PHDs, pVHL and O_2_, other factors can modulate HIF-1*α* level and HIF-1 transcriptional activity in pVHL- and/or O_2_-independent manners. For example, p53 contains hypoxia-induced HIF-1*α* accumulation by promoting Mdm2-mediated ubiquitination and proteasomal degradation 7, which is inhibited by a Jun activation domain-binding protein-1 (Jab1) 8. The molecular chaperone Hsp90 can influence HIF-1*α* degradation 9. The integrity and function of mitochondria are essential to HIF-1*α* accumulation under hypoxic conditions 10,11, and HIF-1*α* acetylation can sensitize the protein to pVHL-mediated ubiquitination and degradation 12. In addition, a variety of small molecules have the capability of inhibiting HIF-1 transcriptional activity, by affecting the synthesis, turnover, heterodimerization, DNA binding, transactivation or signal transduction of the HIF-1*α* 1.

Fatty acid metabolism is connected to signaling transduction networks in various pathogeneses. In cancer, for example, abnormalities in fatty acid metabolism may contribute to the Warburg effect, cachexia, mitochondrial dysfunction, cancer aggressiveness and so on 13. For many catalytic enzymes in fatty acid metabolism, the involvement in cancer development and progression can be partly attributed to the second messenger function of the metabolite. The roles of two key enzymes, cyclooxygenase-2 (COX-2) and 15-lipoxygenase-1 (15-LO1), in carcinogenesis are intriguing, since they appear to function differently in the carcinogenesis of colorectal cancer 14. Following our prior report that prostaglandin E_2_ (PGE_2_) could induce HIF-1*α* synthesis and that the inhibition of COX-2 could suppress HIF-1*α* and HIF-1 transcriptional activity 15, we sought to define the role of 15-LO1 in the regulation of the HIF-1*α*/HIF-1 pathway. This study shows that in opposition to the COX-2 enzyme, 15-LO1 promotes HIF-1*α* turnover and thus suppresses HIF-1 transcriptional activity. The antagonistic modulation of HIF-1*α* by 15-LO1 versus COX-2 would be an excellent experimental model for investigating the modulation of fatty acid metabolism on cancer development and progression.

## Material and Methods

### Cells and culture conditions

Human prostate cancer PC-3 cell line and HEK293 cell line were purchased from American Cell Type Collection (Manassas, VA). For hypoxic exposure (1% O_2_), cells were placed in a sealed modular incubator chamber (Billups-Rothenberg, Del Mar, CA) flushed with a gas mixture containing 1% O_2_, 5% CO_2_, and balanced with N_2_.

### Antibodies and reagents

Monoclonal anti-HIF-1*α* and anti-HIF-1*β* antibodies were from BD Transduction Laboratories (San Jose, CA) or Novus Biologicals (Littleton, CO), respectively. Anti-human recombinant 15-LO1 antibody 16 was a generous gift from Dr. Sigal at the University of California at San Francisco. The polyclonal antibodies against ubiquitin (FL-76) and actin, and monoclonal antibody against Gal4 (DBD; RK5C1) were from Santa Cruz (Dallas, TX). Monoclonal anti-Flag (M2) antibody was from Sigma-Aldrich (St. Louis, MO). Monoclonal anti-pVHL antibody was from BD Pharmingen (San Jos, CA). Polyclonal human anti-human topoisomerase I (TOPO-I) antibody was from *Topo*GEN (Port Orange, FL). Polyclonal anti-SP-1 antibody was from Geneka Biotechnology (Montreal, Que). Linoleic acid was from Cayman Chemical (Ann Arbor, MI), and dissolved in dimethyl sulfoxide immediately after ethanol solvent was evaporated under a gentle stream of nitrogen. Caffeic acid (CA) and cycloheximide (CHX) were from Biomol International (Plymouth, PA). PD146176 (PD) was from Parke-Davis Pharmaceutical Research (Ann Arbor, MI). Dimethyloxalyglycine (DMOG) was from Frontier Scientific (Logan, UT).

### Plasmids

Plasmids containing a 2707 base pair full-length human 15-LO1 cDNA either in sense (pcDNA3.1/15-LOS) or antisense (pcDNA3.1/15-LOAS) were previously described 17. These plasmids were used in transfection to PC-3 cells with the GenePorter reagent following the manufacturer's recommended protocol (Gene Therapy Systems, San Diego, CA). Stably transfected clones were selected with G418 (400 *μ*g/mL).

For mutagenesis, the pcDNA3.1/15-LOS was used as a template in polymerase chain reaction (PCR). Chimeric primers synthesized were A (5′-ACGTGCGGCCGCGatg ggtctctaccgcatcc-3′, introducing a 5′ *Not* I site), B (5′-ACGTGCGGCCGCG**ATG**accggccgcactgtgggcgaggac-3′, introducing 5′ *Not* I and a ATG codon), E (5′-CAGTGAA TTCttagatggccacactgttttccacc-3′, introducing 3′ *Eco*R I site), F (5′-ggaaattaacgtcc**T**ggccaggactggg-3′, introducing G to T mutation in nucleotide 1208), and G (5′-cccagtcctggcc**A**ggacgttaatttcc-3′, introducing C to A mutation in nucleotide 1208). To make an AE construct containing the wild-type 15-LO1, nucleotide 4–1968 of the coding region was amplified with primers A and E. To make a BE construct of truncated 15-LO1 lacking *β*-barrel domain, nucleotide 337–1968 was amplified with primers of B and E. To make an AGFE construct containing a 15-LO1 with Arg^402^ → Leu mutation, two parts of the coding region were amplified with primer pairs of A and G, and F and E, respectively. The two fragments were annealed as template and amplified into a single product by primers A and E. The final PCR products were cloned into pcDNA3.1 following appropriate restriction digest, and were confirmed by DNA sequencing analysis with nested primers.

Two HIF-1*α* expressing plasmids, Flag/HIF-1*α* and pcDNA3.1/HIF-1*α*, were constructed by using p3xFLAG-myc-CMV-25 (Sigma-Aldrich) and pcDNA3.1 (Invitrogen, Carlsbad, CA) mammalian expression vectors, respectively, with the human HIF-1*α* coding sequence inserted into the *Hin*dIII/*Not*I site. Construction of the plasmids HA-Gal4-HIF-1*α* ODD (530–652) and HA-Gal4-HIF-1*α* ODD (P564A) was as previously described 18. Firefly luciferase expressing plasmids used in the study were previously described as well 15. Reporter plasmid pBI-GL V6L contains hypoxia response element (HRE) derived from the promoter of *VEGF* gene, while p2.1 contains a 68-bp HRE from the *ENO1* gene. Control reporter plasmid pTK-RL expressing *Renilla* luciferase was from Promega (Madison, WI).

### Protein isolation and Western blot analysis

To isolate nuclear proteins, cells were washed with cold phosphate buffered saline (PBS) and recovered by centrifugation at 500*g* for 5 min at 4°C. Crude nuclear extracts were prepared by resuspending cell pellets in an ice-cold buffer containing 10 mmol/L Tris-HCl, pH 7.5, 1.5 mmol/L MgCl_2_, and 10 mmol/L KCl with 2 mmol/L dithiothreitol (DTT), 0.4 mmol/L phenylmethylsulfonyl fluoride, 2 *μ*g/mL leupeptin, 2 *μ*g/mL aprotonin, 2 *μ*g/mL pepstatin, and 1 mmol/L Na_3_VO_4_, for 10 min on ice. Nucleus pellets were collected by centrifugation at 17,000*g* for 10 min at 4°C, and supernatant was saved as cytoplasmic fraction. The nuclei were resuspended in ice-cold buffer containing 0.5 mol/L NaCl, 20 mmol/L Tris, pH 7.5, 20% (v/v) glycerol and 1.5 mmol/L MgCl_2_ with the cocktail of protease and phosphatase inhibitors, and incubated on a rotator for 30 min at 4°C, before the nuclear protein was harvested by centrifugation at 20,000 *g* for 30 min at 4°C. To prepare for whole cell lysates, cells were lysed in light protected buffer containing 100 mmol/L potassium phosphate, pH 7.8, and 0.2% (v/v) Triton X-100 supplemented with 2 mmol/L DTT. Protein concentration was determined by the BCA method (Pierce, Rockford, IL). Equal amounts of protein (30 *μ*g) from each sample were used in Western blotting.

### Enzyme-linked immunosorbent assay

VEGF was measured by enzyme-linked immunosorbent assay (ELISA) kit from R&D (Minneapolis, MN). Cells seeded onto 6-well plates at a density of 4 × 10^4^ cells per well were incubated for 24 h in complete media, and subjected to treatments in triplicate. Subsequent media were collected for ELISA, while cells were harvested for cell number counting with a TC2 automated cell counter (Bio-Rad, Hercules, CA). Triplicate measurements were made for each sample.

### Transient transfection and reporter gene assay

Cells at 75% confluence in six-well plates were transfected in triplicate with the GenePorter Reagent (Gene Therapy Systems). Transfected HEK293 cells for immunoprecipitation were grown in 10-cm culture dishes. For luciferase reporter assays in six-well plates, each well of cells was transfected with 0.1 *μ*g DNA of reporter plasmid pBI-GL V6L or p2.1, and each well of PC-3 cells was transfected with 1 *μ*g reporter plasmid DNA. Control reporter plasmid pTK-RL was used in 0.05 *μ*g/well for HEK293 cells and 0.5 *μ*g/well for PC-3 cells. Twenty-four hours after transfection, the cells were subjected to different treatments before harvested. Luciferase activity was determined with the Dual-Luciferase Reporter System (Promega) on a LUMIstar Galaxy luminometer (BMG Labtechnologies, Durham, NC). Relative luciferase activity was documented by normalizing activity of the experimental reporter (*Firefly*) to that of the control (*Renilla*).

### RNA isolation and reverse transcription coupled PCR

Total RNA was isolated from cells grown in 10 cm dishes using TRIzol Reagent (Life Technologies, Carlsbad, CA). A one-step and relative quantitative reverse transcription-PCR (RT-PCR) was performed using the Titan One Tube RT-PCR System (Roche Diagnostics, Indianapolis, IN), in a 50-*μ*L volume containing 0.5 *μ*g of total RNA, 0.2 *μ*mol/L primers and 4 *μ*L of 18S internal standard with an 18S primer pair/competimers ratio of 1:3.5 (Ambion, Austin, TX). Primers for detecting HIF-1*α* were 5′-c ttaagaaggaacctgatgc-3′ and 5′-cttgattgagtgcagggtc-3′. VEGF was detected by primers 5′-tcgggcctccgaaaccat-3′ and 5′-gcgcagagtctcctcttc-3′. The reaction involved an initial incubation at 50°C for 30 min, followed by 30 cycles of 94°C for 30 sec, 60°C for 30 sec and 72°C for 40 sec, with a final elongation at 72°C for 5 min.

### Immunoprecipitation

Cells were washed with cold PBS and lysed on ice in buffer containing 50 mmol/L Tris, pH 7.4, 150 mmol/L NaCl, 1 mmol/L Ethylenediaminetetraacetic acid (EDTA), and 1% (v/v) Triton X-100 supplemented with the protease and phosphatase inhibitor cocktail. After removing cellular debris by centrifugation at 10,000*g* for 10 min at 4°C, lysates were precleared by adding 1.0 *μ*g of control IgG together with 20 *μ*L of resuspended volume of agarose conjugate, and incubated at 4°C for 30 min. For each sample, 1 mg protein was immunoprecipitated with 1 *μ*g primary antibody and 40 *μ*L beads, or with antibody agarose conjugate, at 4°C with rotation for 1.5 h. Immunoprecipitates were washed four times in a buffer of 50 mmol/L Tris, pH 7.4, and 150 mmol/L NaCl, and recovered by centrifugation at 1000*g* at 4°C for 5 min. Immunoprecipitates were washed again in ice-cold PBS, resuspended in 50 *μ*L of 2X SDS buffer, and boiled for 5 min. The 30-*μ*L aliquots were analyzed by SDS-PAGE and Western blotting.

### Ubiquitination assays

For the in vivo ubiquitination assay, cells at 75% confluence on 10-cm dishes were transfected with mammalian expression plasmids. Twenty-four hours after transfection, the cells were subjected to treatments as designed, and were subjected to an additional treatment with 5 *μ*mol/L MG132 for another 4 h. Cells were washed with cold PBS and lysed in cold buffer containing 50 mmol/L Tris, pH 7.4, 150 mmol/L NaCl, 1 mmol/L EDTA, and 1% (v/v) Triton X-100. Immunoprecipitation and Western blotting were performed with appropriate antibodies.

For in vitro ubiquitination assay, HIF-1*α* ODD translates was generated in vitro from the Gal4-HIF-1*α* ODD (530-652) plasmid with the TNT T7 coupled transcription/translation system (Promega), in the presence of 2 *μ*Ci ^35^S-methionine. Briefly, cells were incubated in ice-cold hypotonic buffer (20 mmol/L Tris, pH 7.4, 5 mmol/L MgCl_2_, and 8 mmol/L KCl, with 1 mmol/L DTT and plus inhibitor cocktail) for 15 min, and were subjected to three cycles of freeze and thaw. After centrifugation at 14,000*g* for 5 min, the supernatant was ultra centrifuged at 100,000*g* for 4 h, and was aliquoted and stored at −80°C. ^35^S-labeled translates (2 *μ*L) were incubated in the presence of S100 extracts (100 *μ*g) supplemented with 8 *μ*g/*μ*L ubiquitin (Sigma-Aldrich), 100 ng/*μ*L ubiquitin aldehyde (BostonBiochem, Cambridge, MA) and energy-regenerating system (20 mmol/L Tris, pH 7.4, 2 mmol/L ATP, 5 mmol/L MgCl_2_, 40 mmol/L creatine phosphate and 0.5 *μ*g/*μ*L creatine kinase) in a 40-*μ*L volume for 1.5 h at 30°C. Products were immunoprecipitated with anti-Gal4 and resolved by SDS-PAGE.

### Data analysis

**Figure 1:**
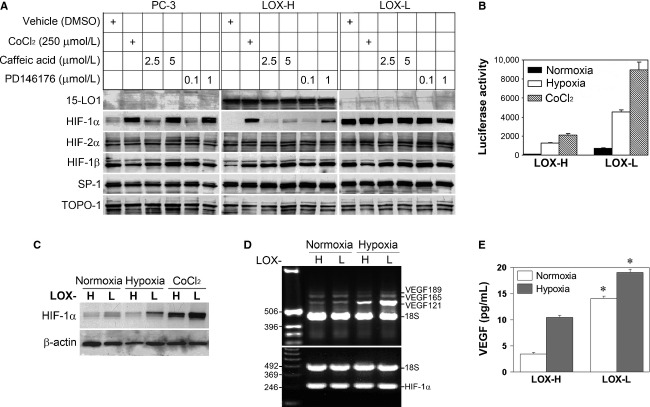
Stable 15-LO1 transfection altered HIF-1*α* and HIF-1 transcriptional activity. (A) Western blotting analysis of crude nuclear extracts from PC-3, LOX-H and LOX-L cells. The cells in six-well plates at 70% confluence were subjected to overnight serum starvation, and treated with different reagents in serum-free media for 16 h before harvest. 15-LO1 inhibitors Caffeic acid and PD146176 were dissolved in dimethyl sulfoxide (DMSO). DMSO volumes were <0.5% (v/v) in culture medium. Immunoblots were repeatedly stripped and probed. (B) Transient transfection and reporter gene assays were conducted in LOX-H and LOX-L cells. After transfection for 24 h, cells were cultured for additional 16 h under normoxic or hypoxic conditions, or treated with CoCl_2,_ before harvest. The figure represents mean ± SD of triplicate of one experiment. (C) Whole cell lysates in the transient transfection assay in B were analyzed for HIF-1*α* expression by Western blot. Similar results were shown in triplicate of one experiment, and reproduced in two more repeats. (D) Total RNA in LOX-H and LOX-L cells was analyzed for transcription of the VEGF (upper panel) and *HIF-1α* (lower panel) gene by RT-PCR after cells were subjected to normoxia or hypoxia for 24 h. DNA standards and the products of major VEGF isoforms are indicated. (E) Culture medium in experiment D was assayed for VEGF production with ELISA. Bars indicate standard deviations of triplicates and asterisk (*) denotes statistical significance compared to those in LOX-H cells (*P* < 0.05).

Experiments presented in the figures were representative of reproducible experiments of 2 or more. Data of luciferase activity were the average of triplicates. Densitometry quantification in Figure 3 was performed using the Bio-Rad image analysis system. Statistical analysis in Figure 1E was performed using a 2-sample *t* test. A *P* < 0.05 was considered statistically significant.

## Results

### HIF-1*α* and HIF-1 transcriptional activity are modulated in cells with differential 15-LO1 expression

Forced expression of 15-LO1 by stable transfection in human prostate cancer PC-3 cells has been previously reported 17, resulting in a marked increase in 15-LO1 enzymatic activity. We transfected PC-3 cells with the same expression constructs to isolate stably transfected clones. Transfection with 15-LO1 coding sequence in sense orientation resulted in LOX-H clones with 15-LO1 overexpression, while transfection with the same sequence in antisense orientation led to the isolation of LOX-L clones that showed decreased 15-LO1 levels relative to the control clones. Following confirmation of the 15-LO1 expression by Western blotting, the same blots were used to examine the effect of 15-LO1 on HIF-1*α* level.

Under standard culture conditions (21% O_2_, normoxia), basal HIF-1*α* levels were consistently lower in LOX-H cells and higher in LOX-L cells as compared with PC-3 cell control clones that expressed a constitutive level of HIF-1*α* (Fig. 1A). The differences existed when cells were treated with CoCl_2_, which inhibits HIF-1*α* proteasomal degradation. Other proteins, such as HIF-2*α*, HIF-1*β*, SP-1 and TOPO-1, did not present similar dynamics. Two 15-LO1 inhibitors, CA 19 and PD146176 (PD) 20, increased HIF-1*α* in a dose-dependent fashion in PC-3 control cells that express a minimal level of 15-LO1 activity, and in LOX-H cells that express a high level of 15-LO1 activity. The observed HIF-1*α* inhibition seemed to be mediated by enzymatic activity of the 15-LO1.

Because 15-LO1 overexpression and inhibition resulted in different HIF-1*α* levels, representing a novel mechanism by which lipid metabolism modulates HIF-1 signaling, we next investigated biologic significance of the 15-LO1 modulation by measuring HIF-1 transcriptional activity and HIF-1 downstream gene (*VEGF*) expression. We found that HIF-1*α* -dependent transcriptional activity as shown in luciferase assays was markedly higher in LOX-L cells than in LOX-H cells not only under normoxia but also under hypoxia (1% O_2_) or in cells treated by CoCl_2_ (Fig. 1B). The differential luciferase activity was in full agreement with the changes in HIF-1*α* level in those cells (Fig. 1C). The inhibitory effect of 15-LO1 on HIF-1 was confirmed by alternative methods. As demonstrated by RT-PCR analysis, the differences in *VEGF* gene expression at mRNA level existed between LOX-H and LOX-L cells, where hypoxia induced VEGF121 to a greater extent than the other isoforms (Fig. 1D) in agreement with previous findings 21. The differences in *VEGF* expression between LOX-H and LOX-L cells were also detected at translational level. Determined by ELISA, VEGF secretion was consistently lower in LOX-H cells compared to LOX-L cells under normoxia or hypoxia with statistically significant differences (Fig. 1E). Above results indicate that forced overexpression of 15-LO1 decreases HIF-1*α* with a resultant reduction in HIF-1 transcriptional activity and HIF-1 target *VEGF* gene expression, whereas inhibition of 15-LO1 does the opposite, and furthermore, 15-LO1 enzyme activity is likely involved in the process.

### Attenuation of 15-LO1 restores functional HIF-1*α*

**Figure 2:**
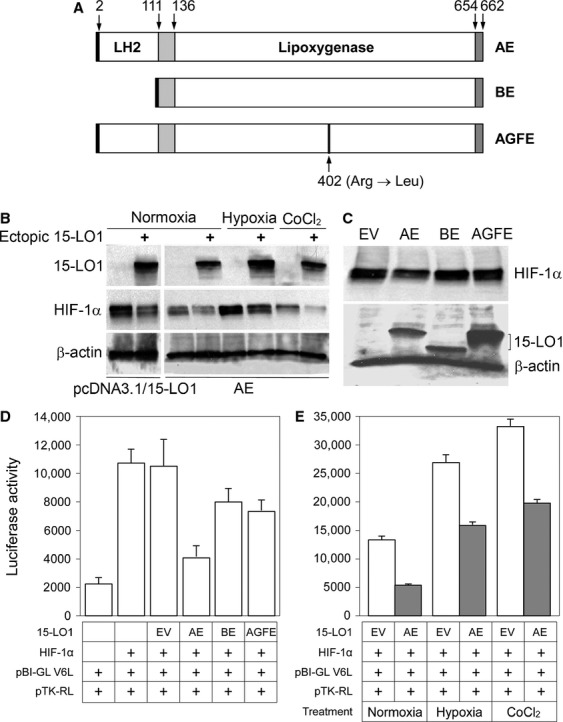
Transient 15-LO1 transfection altered HIF-1*α* and HIF-1 transcriptional activity. (A) Structural diagram of 15-LO1 mutagenesis. The AE construct contains wild-type 15-LO1. The BE construct contains an N-terminal *β*-barrel domain truncation mutant. The AGFE construct contains Arg^402^ → Leu replacement. Arrows indicate the position of the amino acids. (B) Western blot showing effect of wild-type 15-LO1 overexpression (upper panel) in either pcDNA3.1 (pcDNA3.1/15-LO1) or p3×FLAG-myc-CMV-25 (AE) constructs on HIF-1*α* level (middle panel). The lower panel is the loading control shown by *β*-actin expression. (C) Western blot showing the expression of wild-type 15-LO1 and its mutants (BE and AGFE) on HIF-1*α* level (upper panel) under normoxic conditions. EV: control transfection with empty vector. In lower panel, the blot was reprobed for the transfected 15-LO1 mutants. (D) VEGF-driven luciferase reporter in pBI-GL V6L was used to assess functional significance of the HIF-1*α* inhibition. Expression constructs used in the transient co-transfection are indicated below the histogram. A representative result with HEP293 cells under normoxia is shown. (E) The same experimental conditions were used to assess the HIF-1*α* inhibition in hypoxia and following CoCl_2_ treatment. Relative luciferase activity was normalized by the activity of *Firefly* luciferase to *Renilla* luciferase in 1.0 *μ*g protein. Bars indicate deviations of triplicates.

To further establish the role of 15-LO1 in regulating HIF-1*α* expression, the site-directed mutagenesis or truncation of human 15-LO1 was carried out to attenuate 15-LO1 function. Two sites that are critical for 15-LO1 function were targeted: Arg^402^ in the C-terminal catalytic domain 22 and *β*-barrel domain in the N-terminal 23. An alternative mammalian expression vector (p3×FLAG-myc-CMV-25) was used in constructing the mutants. Generated expression constructs included a wild-type control with the 1989 bp cDNA covering the full-length of the 15-LO1 coding sequence (the AE construct), a mutant with *β*-barrel domain truncation (the BE construct), and another carrying Arg^402^ → Leu replacement (the AGFE construct) (Fig. 2A).

In transient transfection assays, the AE (wild-type 15-LO1) inhibited co-expressed HIF-1*α* levels in HEK-293 cells (Fig. 2B), and the inhibition was in a similar fashion as that observed in PC-3 cells with stable 15-LO1 overexpression that was stably transfected with the original expression construct carrying the 2707 bp cDNA in a different mammalian expression vector, pcDNA3.1. Importantly, transfection with the mutants, BE or AGFE, failed to decrease HIF-1*α* as compared with the parallel transfection with the AE (Fig. 2C). Consistent with the HIF-1*α* level, HIF-1 transcriptional activity was dynamically modulated among these constructs (Fig. 2D and E). Accordingly, co-transfection of the AE with luciferase reporter to HEK-293 cells resulted in marked inhibition of the reporter gene activity under different conditions (Fig. 2E). In comparison, the inhibitory effect was markedly compromised when either the BE or AGFE mutant was applied (Fig. 2D). These results not only confirm the inhibitory effect of 15-LO1 on HIF-1*α* but also indicate that the functional structures of 15-LO1 are critical for the enzyme to exert the inhibitory effect.

### Inhibition of 15-LO1 activity decreases the rate of HIF-1*α* degradation

HIF-1*α* could be modulated at several levels 1. In order to determine whether or not the modulation by 15-LO1 occurred at transcriptional level, we analyzed HIF-1*α* mRNA levels by RT-PCR in LOX-H and LOX-L cells. HIF-1*α* mRNA expression showed no marked differences between the two cell types under normoxic or hypoxic conditions (Fig. 1D, lower panel), and no differences between LOX-H cells treated with and without 15-LO1 inhibitors (data not shown). These results suggest that the modulation is not at transcriptional level. We next examined whether the modulation took place at posttranslational level, by determining the rate of HIF-1*α* decay. PC-3 cells are known to express a low basal level of 15-LO1 17 and relatively high basal level of HIF-1*α* 15, facilitating a convenient tracking of degradation.

**Figure 3:**
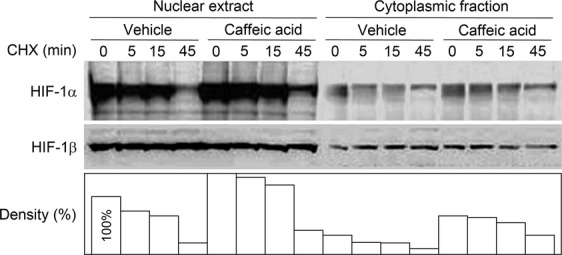
Inhibition of 15-LO1 activity decreases the rate of HIF-1*α* degradation. Western blotting analysis of HIF-1*α* decay in PC-3 cells with or without 15-LO1 inhibitor caffeic acid. Cells were treated with caffeic acid for 22 h under normoxia and then added with CHX (100 *μ*mol/L) for the indicated time. Histogram at the bottom is the quantification of relative HIF-1*α* level.

PC-3 cells pretreated with 15-LO1 inhibitor CA for 22 h were blocked for protein synthesis by cycloheximide (CHX). Nuclear extracts and cytoplasmic fractions from the treated cells were subjected to HIF-1*α* decay analysis. Compared to the vehicle control, PC-3 cells treated with CA contained elevated levels of HIF-1*α* both in the nuclear and cytoplasmic compartments (Fig. 3). Importantly, the rate of HIF-1*α* degradation appeared to be slower in the presence of CA treatment. The effect of CA was specific to the HIF-1*α* subunit, as HIF-1*β* remained constant throughout the study period. These results indicate that the enzymatic activity of 15-LO1 exerts an inhibitory effect specifically on the HIF-1*α* subunit, likely by accelerating its degradation.

### 15-LO1 promotes HIF-1*α* ubiquitination and proteasomal degradation in normoxia

**Figure 4:**
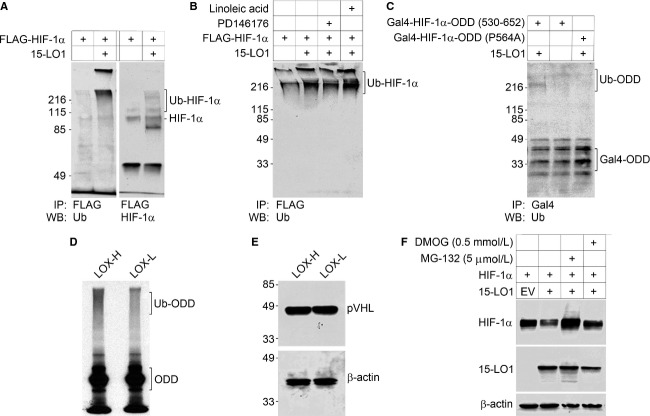
15-LO1 promotes the ubiquitination and degradation of HIF-1*α* in normoxia. Both in vivo and in vitro ubiquitination assays were used to elucidate the mechanism of 15-LO1 mediated HIF-1*α* inhibition. Cells in all experiments were cultured under normoxia (20% O_2_). A series of in vivo ubiquitination assay were conducted in HEK293 cells (A–C). (A) The effect of 15-LO1 on HIF-1*α* ubiquitination (Ub-HIF-1*α*) was detected by immunoprecipitation. (B) The effect of 15-LO1 on HIF-1*α* ubiquitination in the presence of 15-LO1 inhibitor PD146176 and 15-LO1 substrate linoleic acid. (C) The effect of 15-LO1-induced ubiquitination of the HIF-1*α* with either wild-type ODD (530-652) domain or ODD domain mutant (P564A). The ubiquitination of HIF-1*α* polypeptide with wild-type ODD domain was detected as increased Ub-ODD fraction. At the front, fast migrating Gal4-ODD fraction represents un-ubiquitinated ODD. (D) In an in vitro ubiquitination assay, the ubiquitination of radiolabeled HIF-1*α* ODD polypeptide (530-652) was detected in LOX-H or LOX-L cells. (E) Western blot analysis showing pVHL expression in LOX-H and LOX-L cells (Upper panel), *β*-actin levels as loading control (Lower panel). (F) A representative Western blotting analysis of HIF-1*α* expression in HEK293 cells following HIF-1*α* and 15-LO1 co-transfection in the presence of 26S proteasome inhibitor MG132 and PHD inhibitor DMOG.

Protein degradation is a critical regulatory mechanism controlling HIF-1*α* homeostasis 1, mainly through the machinery of unbiquitination-directed proteasomal degradation 1,3,4,18. To investigate whether 15-LO1 could affect this machinery, we conducted in vivo and in vitro ubiquitination assays following previously reported methodology 18. In transient co-transfection in HEK293 cells, 15-LO1 facilitated HIF-1*α* ubiquitination (Fig. 4A), which was attenuated by 15-LO1 inhibitor PD146176, but enhanced by 15-LO1 substrate linoleic acid (Fig. 4B). The results confirm the involvement of 15-LO1 enzymatic activity and suggest potential involvement of 15-LO1 metabolites.

In further studies defining target site of the inhibitory effect, 15-LO1 promoted the ubiquitination of a HIF-1*α* polypeptide containing ODD domain (530–652) (Fig. 4C). The accumulation of ubiquitinated polypeptide was decreased when proline residue Pro^564^ was mutated to alanine (Fig. 4C). Moreover, we confirmed the results by examining ubiquitination rate of in vitro synthesized HIF-1*α*. Proteins synthesized with cell-free transcription coupled translation are mostly in a naïve state, free of modification and degradation. A radiolabeled HIF-1*α* polypeptide containing ODD domain (530–652) was synthesized in vitro and used as ubiquitination target, and S100 proteins from PC-3 derivative clones were used as the source of ubiquitin. In these assays, ubiquitination was consistently more pronounced with the addition of S100 extracts from LOX-H rather than LOX-L cells (Fig. 4D). These results suggest that 15-LO1 promotes HIF-1*α* ubiquitination and that this process requires an intact HIF-1*α* ODD domain containing Pro^564^. The enhanced HIF-1*α* ubiquitination by 15-LO1 was unlikely due to an increasing level of pVHL, since the levels of pVHL expression in LOX-H and LOX-L cells were identical (Fig. 4E). On the other hand, 15-LO1-mediated HIF-1*α* degradation could be blocked by 26S proteasome inhibitor MG132 (Fig. 4F), or in the presence of the PHD inhibitor DMOG. Pro^564^ hydroxylation, ubiquitination, and 26S proteasome system are thus essential for 15-LO1-mediated HIF-1*α* degradation in normoxia.

## Discussion

In this study, we investigated the role of 15-LO1 in modulating HIF-1*α* homeostasis by altering the amount and the enzymatic activity of 15-LO1 in cultured cells. Using multiple molecular methods, we demonstrated that 15-LO1 decreased HIF-1*α* and suppressed HIF-1 transcriptional activity. This study thus unveiled a link between lipid metabolism and transcriptionally controlled hypoxic response, a previously unrecognized regulation between two seemingly independent aspects of cellular physiology. HIF-1*α* inhibition was determined to be at posttranslational level, through promoting ubiquitination and degradation. Since ubiquitin-directed degradation is the major biological thoroughfare to an efficient purge of regulatory proteins following the stress response 24, further investigation is warranted to assess whether 15-LO1 plays a significant role in fine-tuned homeostatic regulation. Most importantly, we were able to show that the enzymatic activity of 15-LO1 was critical in above mentioned inhibitory effects, as the inhibition could be reversed by antagonizing the enzyme, while substrate of 15-LO1 displayed differential inhibitory effects on 15-LO1-mediated HIF-1*α* ubiquitination as demonstrated in Figure 4B. In fact, addition of 15-LO1 substrate linoleic acid to LOX-H cells was also shown to be able to reduce HIF-1*α* (data not shown), indicating the metabolites derived from 15-LO1 enzymatic activity could contribute to the inhibitory process. This work has for the first time offered an exciting strategy for modulating hypoxic response indirectly by pharmacological adjustment of lipid metabolism. 15-LO1 causes HIF-1*α* instability by increasing HIF-1*α* ubiquitination and hence increasing the rate of degradation. Because the S100 fraction from 15-LO1-overexpressing cells was detected with increased ubiquitination activity, the mechanism underlying the inhibitory effect on HIF-1*α* could be complex. Nonetheless, several clues from this study may direct further investigation. First, the enzymatic activity of 15-LO1 is required for inhibition. This is not only supported by changes in HIF-1*α* level in cells with different amounts of 15-LO1 or cells treated with 15-LO1 inhibitors but also supported by mutational assay in which the mutation of Arg^402^ residue in the C-terminal catalytic domain of 15-LO1 results in restoration of HIF-1*α*. The Arg^402^ residue is critical for 15-LO1 enzymatic activity since it is required for substrate binding. Specifically, the activity in the substrate binding of the Arg^402^ → Leu mutant is only 5% of the wild-type activity against arachidonic acid or linoleic acid, which corresponds to the markedly lowered enzymatic reaction rate in the mutant 22. Second, the function of the N-terminal *β*-barrel domain is independently involved, because 15-LO1 with an N-terminal *β*-barrel domain truncation is still able to restore HIF-1*α* in the presence of an intact C-terminal catalytic domain. The N-terminal *β*-barrel domain of 15-LO1 is not thought to be essential for catalytic activity, but is able to mediate intracellular membrane binding 25. The ability to bind intercellular membrane is required for 15-LO1's intracellular organelle degradation function, and this function is important for the removal of aged mitochondria 23,26. Third, the differential HIF-1*α* levels and differential HIF-1 transcriptional activity between cells with forced overexpression of 15-LO1 (LOX-H cells) and cells with 15-LO1 knockdown (LOX-L cells) can be seen in both normoxic and hypoxic conditions, and in cells treated by CoCl_2_. This observation suggests that 15-LO1 may be capable of inducing HIF-1*α* turnover through additional mechanisms that are different from the classic pathway, O_2_-mediated HIF-1*α* ubiquitination/degradation. It is the authors' opinion that 15-LO1 may exert an affect as a co-substrate or an enhancer in the process of HIF-1*α* degradation even under low oxygen tension or in the presence of CoCl_2._ The intracellular organelle degradation capability of 15-LO1, particularly degradation of mitochondria 23,26, is likely to play a role in the reduction in HIF-1*α* in LOX-H cells in such a situation since intact and functional mitochondria are required for HIF-1*α* accumulation under hypoxia 10,11. In addition, direct physical interaction between 15-LO1 and HIF-1*α* was not identified in our experiments (data not shown). The effects of 15-LO1 on reducing HIF-1*α* expression and HIF-1 transcriptional activity are not limited to the prostate cancer PC-3 cells. In a colon cancer cell line HCT116 with forced 15-LO1 stable expression (generous gift from Dr. Thomas Eling of NIEHS, NIH), similar results were demonstrated and were consistently reproducible (data not shown). Further investigations are warranted to understand the more detailed molecular mechanisms.

Studies from past decades have linked oxidative metabolism of polyunsaturated fatty acids to a variety of pathogeneses, including tumorigenesis. Two major classes of enzymes are involved in this process, cyclooxygenase, and lipoxygenase. Many years ago it was proposed that there exists a balance between the procarcinogenic role of the arachidonic acid/COX_2_/PGE_2_ pathway and the anticarcinogenic role of the linoleic acid/15-LO1/13-S-HODE pathway in colonic carcinogenesis 14. Recently, the same group has further demonstrated the tumor suppressor function of 15-LO1 in transgenic mouse by tissue-specific expression of human 15-LO1 27. Our prior studies have shown that PGE_2_ induces HIF-1*α* while COX-2 inhibitor reduces it 15. In our experimental system, a similar balance seems evident between the COX-2/PGE_2_ pathway and the linoleic acid/15-LO1 pathway in the regulation of HIF-1*α*.

The actual role of 15-LO1 in carcinogenesis, however, remains elusive despite much research effort over the last decade. Both anticarcinogenic and procarcinogenic roles have been proposed 17,27–35. In agreement with its tumor suppressor function, 15-LO1 is downregulated in many human cancer types in comparison with their benign counterparts 36–40, whereas it is overexpressed in prostate cancer and its precursors 41. Similarly to its role in colorectal carcinogenesis 27, 15-LO1 appears to function as a tumor suppressor in many other organ systems. For example, overexpression of 15-LO1 inhibits tumor formation and metastasis in breast or lung cancer transgenic mice models 30, and it significantly prolongs survival in rats with glioma via inducing lipid peroxidation 32. In contrast, 15-LO1 appears to promote tumorigenesis and tumor progression in prostate cancer 17,33,34 and malignant melanoma 35. Regarding the role of 15-LO1 in VEGF regulation and angiogenesis, results are also controversial. Overexpression of 15-LO1 in PC-3 prostate cancer cells increases angiogenesis and VEGF secretion in xenografts 17, which is contradictory to results found in this in vitro study based on the same prostate cancer cell line. In nontumor models, Yao et al. have recently found that the 15-LO1 metabolite, 15-HETE, can induce HIF-1*α* expression and HIF-1 transcriptional activity under both normoxic and hypoxic conditions 42. However, 15-LO1 has been demonstrated to exert an antiangiogenic effect by inhibiting VEGF-A expression in rabbit skeletal muscle 43, mouse ischemic retinopathy 44, and hypoxia-induced retinal microvascular endothelial cells 45. Taken altogether, the actual role of 15-LO1 in angiogenesis and carcinogenesis is more complicated than we thought. It may be both context-dependent and content-dependent. For example, it may be dependent on the environment, species, organ, tissue, cell type, major metabolite, and/or dependent on which substrate (arachidonic acid or linoleic acid), enzyme (cyclooxygenase or lipoxygenase) or isozyme (15-LO1 or other LOs) are dominant in the cells and microenvironments.

In summary, 15-LO1 is able to promote HIF-1*α* turnover and to suppress VEGF expression in cultured cells based on forced stable overexpression or transient transfection, whereas 15-LO1 inhibition reverses above effects. Taken together, the results from our prior and current studies thus confirm that the linoleic acid/15-LO1 and COX-2/PGE_2_ pathways have different impacts on the regulation of the HIF-1*α*/HIF-1/VEGF system. The oxidative metabolism of polyunsaturated fatty acid is thought to play a critical role in turning on “metabolic switch” in cancer cells 13. Our findings support that some delicate elements of fatty acid metabolism have “Yin” or “Yang” impact on pathogenic angiogenesis or cancerous “angiogenic switch”, implying potential therapeutic and preventive applications that target angiogenesis through finely tuned fatty acid metabolic microenvironments.
